# Predicting the rupture status of small middle cerebral artery aneurysms using random forest modeling

**DOI:** 10.3389/fneur.2022.921404

**Published:** 2022-07-28

**Authors:** Jiafeng Zhou, Nengzhi Xia, Qiong Li, Kuikui Zheng, Xiufen Jia, Hao Wang, Bing Zhao, Jinjin Liu, Yunjun Yang, Yongchun Chen

**Affiliations:** ^1^Department of Radiology, The First Affiliated Hospital of Wenzhou Medical University, Wenzhou, China; ^2^Department of Radiology, Wenzhou Central Hospital, Wenzhou, China; ^3^Department of Neurosurgery, Renji Hospital Shanghai Jiaotong University School of Medicine, Shanghai, China

**Keywords:** middle cerebral artery, rupture, random forest, small aneurysm, morphology

## Abstract

**Objective:**

Small intracranial aneurysms are increasingly being detected; however, a prediction model for their rupture is rare. Random forest modeling was used to predict the rupture status of small middle cerebral artery (MCA) aneurysms with morphological features.

**Methods:**

From January 2009 to June 2020, we retrospectively reviewed patients with small MCA aneurysms (<7 mm). The aneurysms were randomly split into training (70%) and internal validation (30%) cohorts. Additional independent datasets were used for the external validation of 78 small MCA aneurysms from another four hospitals. Aneurysm morphology was determined using computed tomography angiography (CTA). Prediction models were developed using the random forest and multivariate logistic regression.

**Results:**

A total of 426 consecutive patients with 454 small MCA aneurysms (<7 mm) were included. A multivariate logistic regression analysis showed that size ratio (SR), aspect ratio (AR), and daughter dome were associated with aneurysm rupture, whereas aneurysm angle and multiplicity were inversely associated with aneurysm rupture. The areas under the receiver operating characteristic (ROC) curves (AUCs) of random forest models using the five independent risk factors in the training, internal validation, and external validation cohorts were 0.922, 0.889, and 0.92, respectively. The random forest model outperformed the logistic regression model (*p* = 0.048). A nomogram was developed to assess the rupture of small MCA aneurysms.

**Conclusion:**

Random forest modeling is a good tool for evaluating the rupture status of small MCA aneurysms and may be considered for the management of small aneurysms.

## Introduction

Unruptured aneurysms have been increasingly detected with the development of computed tomography angiography (CTA) and magnetic resonance angiography (1–3). The majority of incidentally detected aneurysms are small (<7 mm) (4, 5). Unruptured small aneurysms are often considered stable and are recommended for conservative treatment with imaging surveillance (6–8). However, recent reports have found that the proportion of small aneurysms in patients with subarachnoid hemorrhage (SAH) was considerable; 75% of ruptured aneurysms were <7 mm (9). To avoid the consequences of SAH, an increasing number of novel preventive treatments have been applied for small unruptured aneurysms (10, 11). All these contradictions make the treatment of patients with unruptured small aneurysms controversial. Therefore, a novel methodology is necessary to construct a rupture prediction model for small aneurysms to facilitate clinical decisions. Recently, machine learning (ML) has been used to classify aneurysm rupture (12–14). It could not only detect important relationships of the risk factors for aneurysm rupture but could also be simply and rapidly applied to make predictions (12–14). Random forest, an important ML tool for prediction and risk analysis, has been widely used because of its good performance and relatively high accuracy (14–16). Xia et al. (17) showed that the random forest model achieved good performance in predicting the clinical outcome after rupture of anterior communicating artery aneurysms with areas under the receiver operating characteristic (ROC) curve (AUC) of 0.90 in the internal test and 0.84 in the external test. Lv et al. (18) found that a user-friendly nomogram incorporating clinical factors and scoring systems could be convenient for predicting mortality and facilitating physician decision-making. Aneurysm morphologies, such as size, size ratio (SR), aspect ratio (AR), and irregular shape have been reported as significant risk factors for aneurysm rupture (12, 19–21). However, the application of ML for predicting the rupture of small aneurysms in specific locations has not been reported.

This study aimed to develop a random forest model to predict the rupture status of small middle cerebral artery (MCA) aneurysms. In addition, we developed an easy and visualized nomogram to facilitate clinical application.

## Materials and methods

### Patient selection

**Figure 1:**
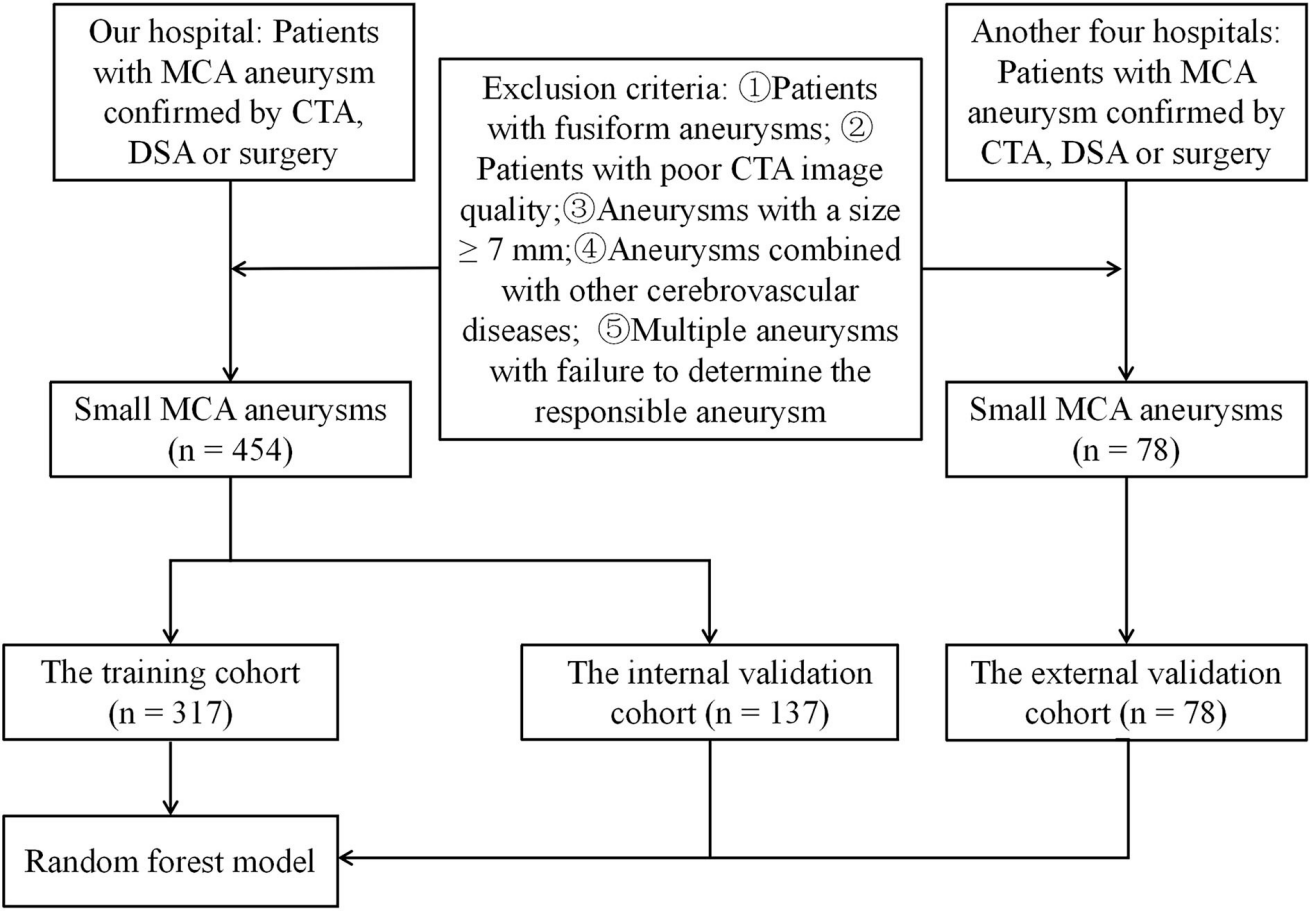
The flowchart of this study.

This study was approved by our institutional ethics committee, which waived the requirement for written informed consent. Between January 2009 and June 2020, 426 consecutive patients with 454 small MCA aneurysms detected using CTA in a hospital were enrolled in this study. The MCA aneurysms with a diameter <7 mm were defined as small. A ruptured aneurysm is defined as a plain CT scan or cerebrospinal fluid examination showing SAH that is confirmed by CTA, digital subtraction angiography, or surgery (21). The exclusion criteria were as follows: patients with fusiform aneurysms, poor CTA image quality, aneurysms with a size ≥ 7 mm, aneurysms combined with other cerebrovascular diseases (such as, Moyamoya disease or arteriovenous malformations), and multiple aneurysms with failure to determine the responsible aneurysm. The flowchart of the study is shown in Figure 1. All aneurysms were randomly divided into the training and validation cohorts (*n* = 7:3). Additional independent datasets were used for external validation from four other hospitals (B, C, D, and E): hospital B (from September 2019 to March 2020), hospital C (from January 2017 to October 2019), hospital D (from January 2018 to June 2021), and hospital E (from January 2018 to June 2021). A total of 78 small MCA aneurysms were included in the final external validation cohort.

### CTA image acquisition

In hospital A, the CTA images were acquired using three CT scanners, including a 320-detector row CT scanner (Aquilion ONE, Toshiba Medical Systems, Japan) with a 0.5 mm section thickness, a 512 × 512 matrix size, a 0.5 mm reconstruction interval, a 100 kV tube voltage, and a 300 mAs tube current; a 64-channel multidetector CT scanner (Lightspeed VCT 64 General Electric Medical Systems, Milwaukee, WI, USA) with a 0.625 mm section thickness, a matrix size of 512 × 512, a 0.625 mm reconstruction interval, a 100 kV tube voltage, and a 500 mAs tube current; and a 16-channel multidetector CT scanner (Lightspeed pro16; General Electric Medical Systems, Milwaukee, Wisconsin, USA) with a 1.25 mm section thickness, a matrix size of 512 × 512, a 1.25 mm reconstruction interval, a 120 kV tube voltage, and a 300 mAs tube current. The CTA imaging protocol has been described in detail previously (22). The details of the CTA image scanning in the other four hospitals are described in Supplemental Digital Content 1.

### Morphological parameters definition

**Figure 2:**
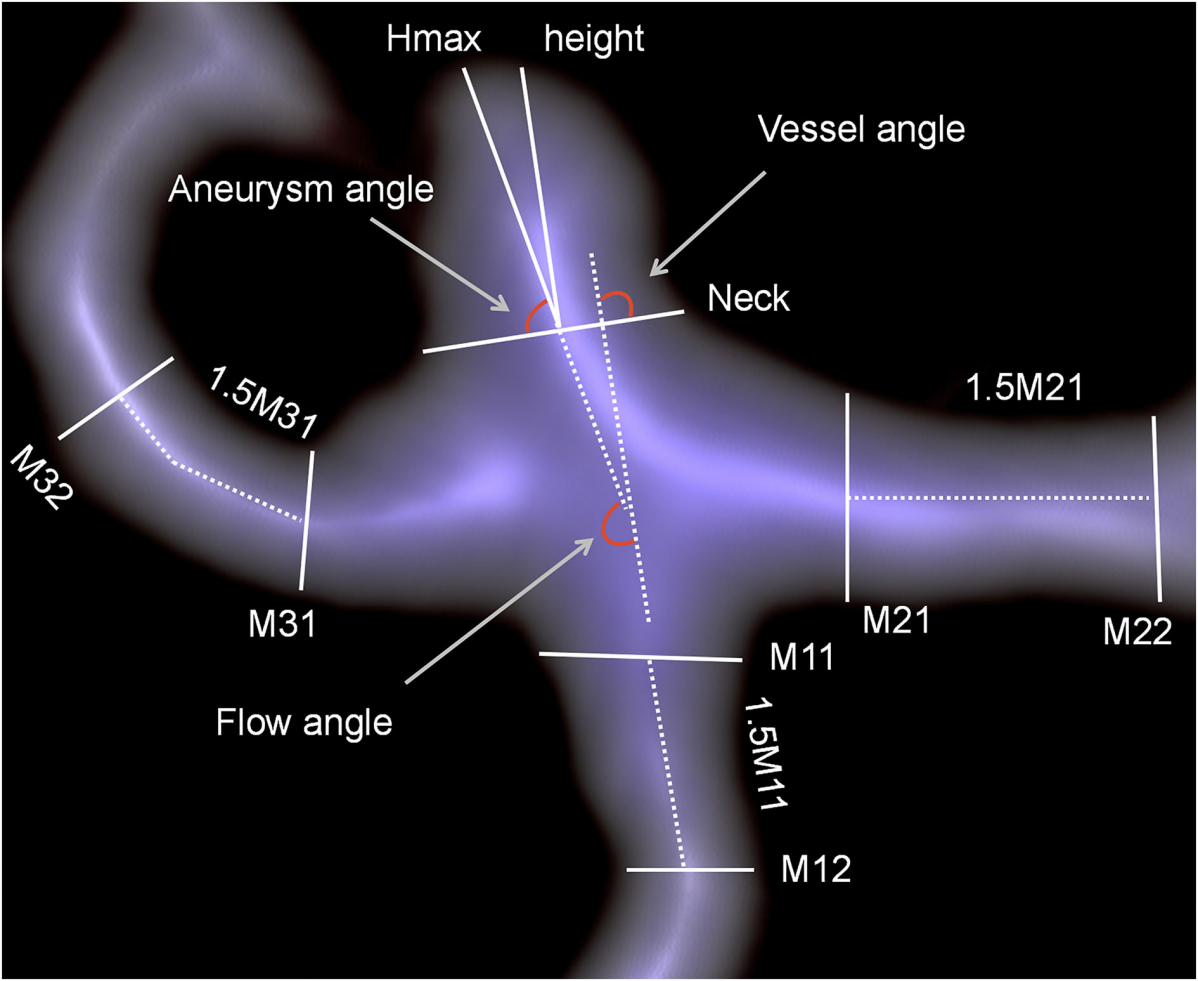
Measurements of aneurysm morphological parameters.

Morphological parameters of the aneurysm, such as aneurysm size, aneurysm height, perpendicular height, neck size, width, vessel size, aneurysm angle, vessel angle, and flow angle, were measured using a CTA image reconstruction workstation (Version 4.6; GE Medical Systems). The measurement of aneurysm morphological parameters has been described in previous studies and is shown in Figure 2 (23). The aneurysm had the largest cross-sectional diameter. The aneurysm height was the greatest distance between the center of the aneurysm neck and the aneurysm dome. Vessel size was defined as the mean of all arteries' vessel diameters compared with the aneurysm. The diameter of a specific artery was determined by averaging the diameter of the cross-section of the vessel next to the aneurysm neck (D1) and the diameter of the cross-section at a 1.5 × D1 distance from the aneurysm neck. The bottleneck ratio was defined as the ratio of aneurysm width to neck size. The AR is the ratio of the perpendicular height to the neck size. The SR is the ratio of aneurysm height to vessel size. The aneurysm angle was the angle formed between the plane of the aneurysm neck and the vector of the aneurysm height. The flow angle was defined as the angle between the aneurysm height line and the vector of blood flow in the parent artery. The vessel angle was defined as the angle between the aneurysm neckline and the blood flow vector. The daughter dome had an irregular protrusion of the aneurysm wall.

### Feature selection and model development

Primary data from hospital A were randomly assigned to the training group (70%, *n* = 317) and the internal validation group (30%, *n* = 137). Feature selection in the training group was performed using univariate and multivariate logistic analyses. The hyperparameters of the random forest model were obtained by a 5-fold cross-validation. The n_estimators, max_depth, and min_samples_split values were 6, 6, and 12, respectively. The performance of the random forest model was evaluated using the AUC, sensitivity, specificity, and overall accuracy. The performance of the model was tested using training and validation cohorts. A nomogram was constructed based on multivariate logistic analysis.

### Statistical analysis

The chi-squared test was used for categorical variables. Student's *t*-test or the Mann–Whitney *U*-test was used for continuous variables between the two groups, and an ANOVA test was used for continuous variables between the three groups. Continuous variables were expressed as mean ± standard deviation (SD), and categorical variables were expressed as frequency (percentage). The DeLong test and the Bonferroni correction were used to compare the AUCs of these models. All statistical analyses were performed using R 3.5.1, Python 3.5.6, and SPSS 23.0 (IBM Corp, Armonk, New, USA). Statistical significance was defined as a two-tailed *p*-value of <0.05.

## Results

### Baseline characteristics

In total, 426 patients with 454 small MCA aneurysms were enrolled in this study. A total of 294 patients with 317 small MCA aneurysms were randomly included in the training cohort, and 132 patients with 137 small MCA aneurysms were randomly selected in the internal validation cohort. Therefore, 78 patients with 78 small MCA aneurysms were included for external validation. Supplemental Digital Content 2 shows the baseline characteristics of the training and internal and external validation cohorts. Only the age was significantly different between the training and external validation cohorts. In the training cohort, 166 patients (56.5%) were women. The median age of the patients was 58.2 ± 12.1 years (range, 20–88 years). There were 164 ruptured and 153 unruptured aneurysms. Patients in the ruptured group were younger (55.4 vs. 61.8 years) and had a lower percentage of hypertension (56.2 vs. 71.8%) than those in the unruptured group. The distribution of patients who smoked (20.5 vs. 20.6%) was similar between the two groups (Supplemental Digital Content 3).

### Morphologic characteristics between ruptured and unruptured small MCA aneurysms

The details of the small MCA aneurysms in the training cohort are presented in Table 1. A univariate logistic analysis revealed that 14 morphological parameters were significantly different between the ruptured and unruptured groups. The results of the multivariate logistic regression analysis are shown in Table 2. The independently significant discriminants were SR [odds ratio (*OR*) 1.774, 95% *CI*: 1.006–3.127; *p* = 0.047], AR (*OR* 7.667, 95% *CI*: 2.697–21.795; *p* < 0.001), aneurysm angle (*OR* 0.980, 95% *CI*: 0.964–0.997; *p* = 0.020), daughter dome (*OR* 4.307, 95% *CI*: 1.630–11.379; *p* = 0.003), and multi aneurysms (*OR* 0.243, 95% *CI*: 0.137–0.433; *p* < 0.001).

**Table 1:** The univariate analysis of morphological features of small middle cerebral artery (MCA) aneurysms in the training cohort.

**Variable**	**Sample**	**Unruptured (*****n*** = **153)**	**Ruptured (*****n*** = **164)**	***P-*** **value**
Multi aneurysms (%)	110	76 (49.67%)	34 (20.73%)	<0.001
Irregular (%)	82	21 (13.73%)	61 (37.20%)	<0.001
Daughter dome (%)	40	6 (3.92%)	34 (20.73%)	<0.001
Aneurysm location (%)				0.185
M1	125	65 (42.48%)	60 (36.59%)	
Mbif	180	80 (52.29%)	100 (60.98%)	
Mdist	12	8 (5.23%)	4 (2.44%)	
Projection in axial (%)				0.306
Anterior	169	76 (49.67%)	93 (56.71%)	
Posterior	55	26 (16.99%)	29 (17.68%)	
Neutral	93	51 (33.33%)	42 (25.61%)	
Projection in coronal (%)				0.801
Superior	105	51 (33.33%)	54 (32.93%)	
Inferior	101	51 (33.33%)	50 (30.49%)	
Neutral	111	51 (33.33%)	60 (36.59%)	
Vessel size (mm)	317	2.41 ± 0.58	2.28 ± 0.49	0.06
Size (mm)	317	4.06 ± 1.34	4.75 ± 1.20	<0.001
Aneurysm height (mm)	317	2.67 ± 1.24	3.68 ± 1.18	<0.001
Perpendicular height (mm)	317	2.34 ± 1.09	3.10 ± 1.07	<0.001
Width (mm)	317	3.22 ± 1.22	3.53 ± 0.99	0.001
Neck size (mm)	317	3.49 ± 1.08	3.21 ± 0.80	0.01
AR	317	0.69 ± 0.32	1.01 ± 0.42	<0.001
SR	317	1.17 ± 0.70	1.73 ± 0.82	<0.001
Bottleneck ratio	317	0.93 ± 0.25	1.14 ± 0.36	<0.001
Height width ratio	317	0.73 ± 0.21	0.89 ± 0.27	<0.001
Aneurysm angle (°)	317	71.42 ± 16.61	65.87 ± 16.53	0.004
Vessel angle (°)	317	49.29 ± 25.22	53.97 ± 26.18	0.106
Flow angle (°)	317	135.63 ± 26.98	135.63 ± 29.81	0.797
Parent daughter angle (°)	317	87.43 ± 29.64	79.66 ± 23.17	0.005

*M1, proximal segment of middle cerebral artery; Mbif, main middle cerebral artery bifurcation; Mdist, distal middle cerebral artery; AR, aspect ratio; and SR, size ratio*.

**Table 2:** The multivariate analysis of morphological features of small middle cerebral artery aneurysms in the training cohort.

**Variables**	**OR**	**95% CI**	***P*** **-value**
SR	1.774	1.006–3.127	0.047
AR	7.667	2.697–21.795	<0.001
Aneurysm angle	0.980	0.964–0.997	0.020
Daughter dome	4.307	1.630–11.379	0.003
Multi aneurysms	0.243	0.137–0.433	<0.001

*OR, odds ratio; CI, confidence interval; AR, aspect ratio; and SR, size ratio*.

### Performances of random forest models

**Figure 3:**
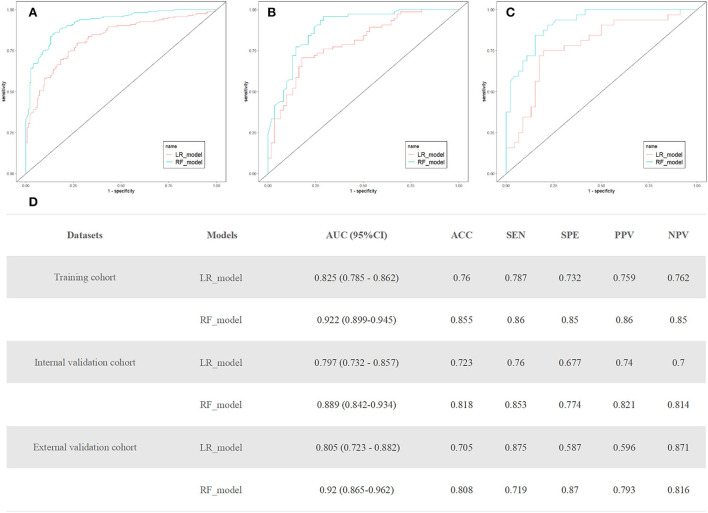
**(A–C)** Receiver operating characteristic (ROC) curves of the random forest and logistic regression models in training, internal, and external validation cohort. **(D)** The performance of the random forest and logistic regression models to predict the rupture of small middle cerebral artery (MCA) aneurysms. AUC, area under the receiver operating curve; CI, confidence interval; ACC, accuracy; SEN, sensitivity; SPE, specificity; PPV, positive predictive value; and NPV, negative predictive value.

The random forest model used five attributes for rupture prediction: SR, AR, aneurysm angle, daughter dome, and multiple aneurysms. Figure 3 shows the prediction performance of the random forest model. The AUCs of the random forest models in the training, internal validation, and external validation cohorts were 0.922 (95% *CI*, 0.899–0.945), 0.889 (95% *CI*, 0.842–0.934), and 0.92 (95% *CI*, 0.865–0.962), respectively. The random forest model outperformed the logistic regression model (*p* = 0.048). The calibration curve of the random forest model for the probability of ruptured small MCA aneurysms demonstrated better agreement between prediction and observation than that of the logistic regression model (Supplemental Digital Content 4).

### Nomogram for predicting rupture risk of small MCA aneurysms

**Figure 4:**
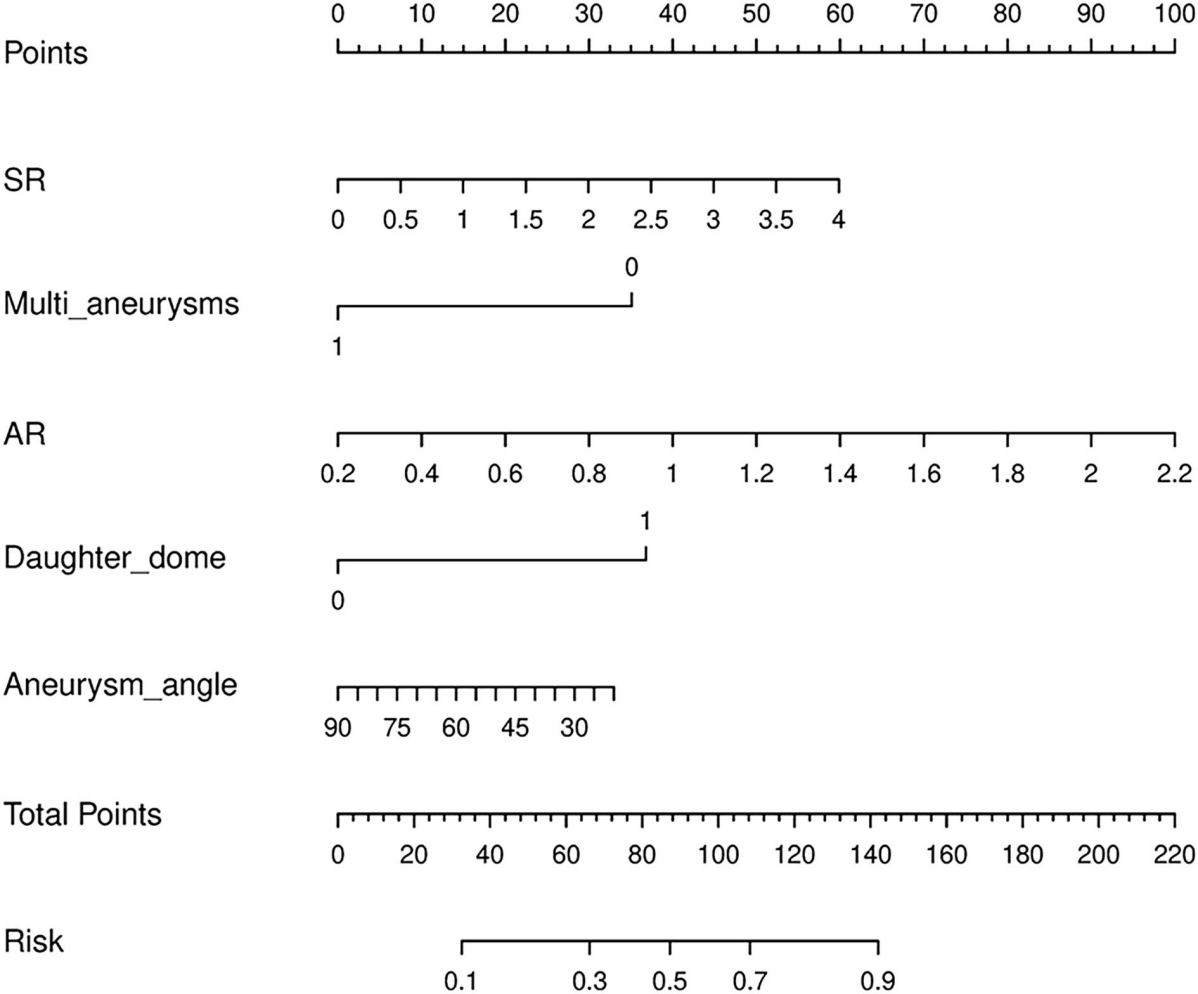
A nomogram for predicting small middle cerebral artery aneurysm rupture. The nomogram incorporated five attributes: SR, multi aneurysms, daughter dome, AR, and aneurysm angle. To use the nomogram, read the scoring points from the “Point” reference line in line with the variable, add the points from all the variables, and find the predicted probability of rupture risk at the bottom “Risk” line. AR, aspect ratio; SR, size ratio.

A logistic regression model that incorporated the above five attributes was also developed and presented as a nomogram (Figure 4). The logistic regression model had satisfactory discrimination ability, with an AUC of 0.825 (95% *CI*, 0.785–0.862), 0.797 (95% *CI*, 0.732–0.857), and 0.805 (95% *CI*, 0.723–0.882) in the training, internal validation, and external validation cohorts, respectively (Figure 3).

## Discussion

In this study, we found that the SR, AR, and daughter dome were associated with aneurysm rupture, whereas aneurysm angle and multiplicity were inversely associated with small MCA aneurysm rupture. The ML method has excellent performance in quantitative individual risk assessments for small MCA aneurysms and may aid in choosing optimal management.

Aneurysm morphology has been reported to be associated with aneurysm rupture (20, 24). Previous studies have shown that SR and AR are consistently associated with aneurysm ruptures (23, 25). A larger SR may increase the area of low aneurysmal wall shear stress and result in more complex flow patterns within the aneurysm (26). These changes may lead to ruptured aneurysms (26). With the increase in AR, the velocity of blood flow in aneurysms slows down, and this hemodynamic change is associated with a higher rupture risk for aneurysms (24). These findings were consistent with those of our studies, which showed that aneurysms with larger SR or AR were more common in ruptured small MCA aneurysms. Another important risk factor for ruptured aneurysms in our study was the presence of a daughter dome. The development of the aneurysm dome may be due to the increased intra-aneurysmal pressure, which increases the risk of aneurysm rupture (23). Moreover, multiple aneurysms are more commonly observed in unruptured small aneurysms (27). We found that aneurysm multiplicity was inversely associated with small MCA aneurysms. Our findings are supported by the current results (28). Therefore, there is a lower risk of small MCA aneurysm rupture in patients with multiple aneurysms than in those with aneurysms in other locations.

In this study, we developed a model to predict the rupture of small MCA aneurysms using five attributes (SR, multiple aneurysms, daughter dome, AR, and aneurysm angle) based on a large dataset. Previous studies have attempted to build a scoring system based on clinical and morphological risk factors to predict the risk of aneurysm rupture. The PHASES score system (29), which was developed from the natural course of unruptured intracranial aneurysms, includes a history of SAH, hypertension status, age, aneurysm size, aneurysm location, and geographical region. Lin et al. (30) analyzed 638 MCA aneurysms and constructed a morphological risk-score model. However, there are distinctive pathophysiological presentations and clinical treatments for large and small intracranial aneurysms (10, 31, 32). Varble et al. (27) developed a model for small aneurysm rupture with an AUC of 0.84 in the training cohort by using the multivariate logistic regression. Apart from location-specific and size-specific intracranial aneurysms, we investigated the use of ML algorithms to assess morphological risk factors for the rupture instability of small MCA intracranial aneurysms and found that the performance of the random forest model was significantly better than that of the logistic regression model. Compared with traditional statistical methods, the ML algorithm-generated model has higher accuracy for aneurysm rupture risk prediction (33) and has become a tool of growing importance in aneurysm detection and stratification (34, 35). Recently, a convolutional neural network was applied to classify the unstable status of 272 patients with small intracranial aneurysms, and this model achieved a sensitivity of 78.76%, a specificity of 72.15%, and an AUC of 0.755 (36). The most important aspect of our study is that we verified our models using internal and external validation datasets, which further verified the robustness and generalizability of the results. We constructed a nomogram based on a logistic regression model and a model visualization figure. The logistic regression model achieved good prediction performance, and the calibration curves of the nomogram demonstrated good agreement between the predicted small MCA aneurysm rupture risk and the actual small MCA aneurysm status.

### Limitations

Although large-scale small MCA aneurysms were analyzed in this study, there are several limitations. First, this was a retrospective study, and selection bias was inevitable. Unruptured aneurysms were incidentally found in hospitalized patients who were generally older and had a history of hypertension. Second, only the morphological features of aneurysms were analyzed in this study; other risk factors, such as hemodynamics, wall enhancement, and genetics, were not included. Third, morphological changes in aneurysms after rupture were not considered in our study. The model predicted only the current rupture status of the aneurysm rather than the future aneurysm risk. Further longitudinal studies are needed to identify whether this model can be used to predict the rupture risk of small aneurysms.

## Conclusion

In summary, we developed a random forest model based on a large number of small MCA aneurysms from multiple centers. The model achieved good prediction performance in both the training and validation cohorts and significantly outperformed the conventional logistic regression model. Moreover, we constructed an easy-to-use nomogram tool for practical applications. Our findings may aid in individualized decision-making for patients with unruptured intracranial aneurysms.

## Data availability statement

The original contributions presented in the study are included in the article/Supplementary material, further inquiries can be directed to the corresponding authors.

## Ethics statement

The studies involving human participants were reviewed and approved by Ethics Committee in Clinical Research of the First Affiliated Hospital of Wenzhou Medical University. Written informed consent for participation was not required for this study in accordance with the national legislation and the institutional requirements.

## Author contributions

JZ drafted the manuscript. JZ, NX, QL, KZ, XJ, HW, BZ, and JL were involved in data collection, verification, and implementation. JZ and YC was involved data analysis. JL and YC critically corrected the manuscript. YY and YC had the idea for the study design. All authors read and approved the final manuscript.

## Funding

This study was supported by the Wenzhou Major Program of Science and Technology Innovation (ZY2020012) and Wenzhou Science and Technology Project (Y2020166 and Y2020164).

## Conflict of interest

The authors declare that the research was conducted in the absence of any commercial or financial relationships that could be construed as a potential conflict of interest.

## Publisher's note

All claims expressed in this article are solely those of the authors and do not necessarily represent those of their affiliated organizations, or those of the publisher, the editors and the reviewers. Any product that may be evaluated in this article, or claim that may be made by its manufacturer, is not guaranteed or endorsed by the publisher.

## Abbreviations

MCA: middle cerebral artery
CTA: computed tomography angiography
AUC: areas under receiver operating characteristic curve
SAH: subarachnoid hemorrhages
ML: machine learning.
