# Supplementary material for: Predicting the rupture status of small middle cerebral artery aneurysms using random forest modeling
Source: Front Neurol. 2022 Jul 28;13:921404. doi: 10.3389/fneur.2022.921404 (PMC9366079; doi:10.3389/fneur.2022.921404)
Supplement: Supplementary file 1 [file Table_1.doc]

**Supplemental Digital Content**

**Supplemental Digital Content 1：The details of CTA image scanning external data information**

1. In hospital B, a 16-channel multidetector CT scanner (uCT510; United Imaging, Shanghai, China) with a 1 mm section thickness, 1024 × 1024 matrix size, 1 mm reconstruction interval, 120 kV tube voltage, and 160 mA tube current was used to acquire the CTA images.
2. In the C hospital, a 320-channel multidetector CT scanner (Aquilion ONE; Toshiba, Tokyo, Japan) with a 0.5 mm section thickness, 512 × 512 matrix size, 0.5 mm reconstruction interval, a 100 kV tube voltage, and a 300 mAs tube current was used to acquire the CTA images.
3. In hospital D, a 256-channel multidetector CT scanner (iCT; Philips Healthcare, Best, The Netherlands) with a 0.9 mm section thickness, 512 × 512 matrix size, a 0.45 mm reconstruction interval, 120 kV tube voltage, and 250 mAs tube current was used to acquire the CTA images.
4. In the E hospital, a 64-channel multidetector CT scanner (SOMATOM Definition AS+; Siemens Healthcare Sector, Florsheim, Germany) with a 0.75 mm section thickness, a 512 × 512 matrix size, a 0.6 mm reconstruction interval, a 120 kV tube voltage, and a 120 mAs tube current was used to acquire the CTA images.

**Supplemental Digital Content 2：Baseline characteristics among the training, internal and external validation cohort.**

| Variable | Sample | Training cohort  (n=294) | Internal validation  cohort  (n=132) | External validation  cohort  (n=78) | P value |
| --- | --- | --- | --- | --- | --- |
| Female/male | 504 | 166/128 | 83/49 | 46/32 | 0.46 |
| Age | 504 | 58.2±12.1^&^ | 59.5±13.1 | 62.7±12.3^&^ | 0.017 |
| Hypertension^a^ | 428 | 156(62.7%) | 69(62.7%) | 43(62.3%) | 0.998 |
| Smoking^b^ | 418 | 51(19.1%) | 21(20.6%) | 18(30.0%) | 0.215 |

a 76/504 (15.1%) missing values.

b 86/504 (17.1%) missing values.

& significant difference between the two groups.

**Supplemental Digital Content 3：Characteristics of demography between ruptured and unruptured patients in the training cohort.**

| Variable | Sample | Unruptured  (n=130) | Ruptured  (n=164) | P value |
| --- | --- | --- | --- | --- |
| Female/male | 294 | 67/63 | 99/65 | 0.129 |
| Age | 294 | 61.8±11.3 | 55.4±12.0 | <0.001 |
| Hypertension^a^ | 249 | 74(71.8%) | 82(56.2%) | 0.012 |
| Smoking^b^ | 248 | 21(20.6%) | 30(20.5%) | 0.994 |

a 45/294 (15.3%) missing values.

b 46/294 (15.6%) missing values.

**Supplemental Digital Content 4：**


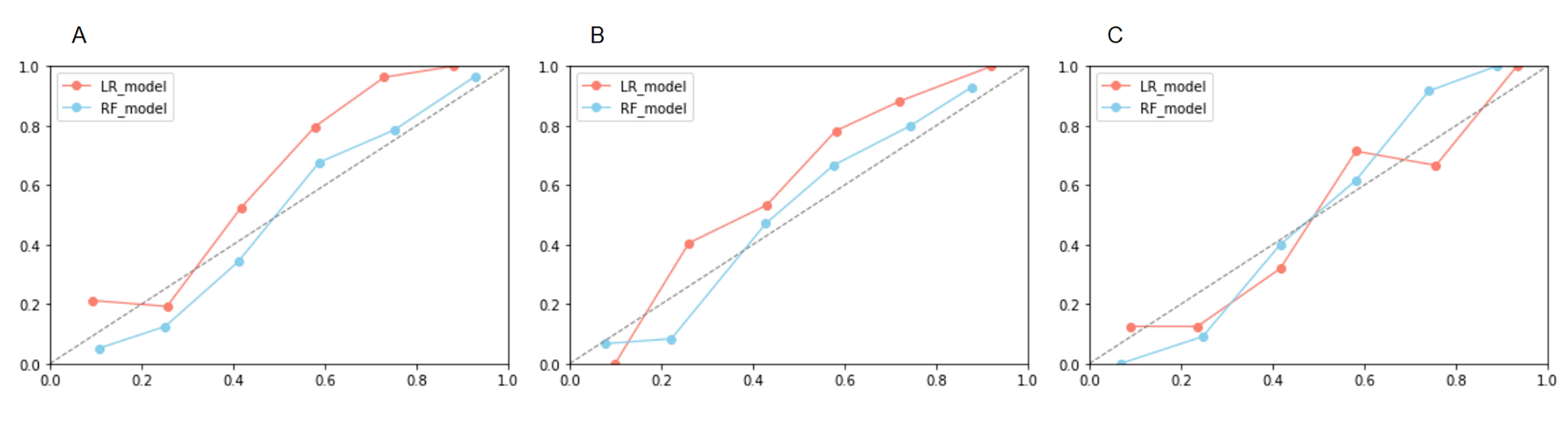


Calibration of the random forest and logistic regression models in the training, internal validation, and external validation cohorts (A-C). Calibration curves depict the calibration of each model in terms of the agreement between the predicted risks of ruptured small middle cerebral artery (MCA) aneurysms and observed outcomes of ruptured MCA aneurysms. The y-axis represents the actual rate of ruptured MCA aneurysms. The x-axis represents the predicted risk of MCA aneurysm rupture. The diagonal dotted line represents perfect prediction using an ideal model. The red and blue lines represent the performance of the logistic regression and random forest, respectively, of which a closer fit to the diagonal dotted line represents a better prediction.
